# Attosecond electronic and nuclear quantum photodynamics of ozone monitored with time and angle resolved photoelectron spectra

**DOI:** 10.1038/srep36613

**Published:** 2016-11-07

**Authors:** Piero Decleva, Nicola Quadri, Aurelie Perveaux, David Lauvergnat, Fabien Gatti, Benjamin Lasorne, Gábor J. Halász, Ágnes Vibók

**Affiliations:** 1 Dipartimento di Scienze Chimiche, Universita’ di Trieste, Via L. Giorgieri 1I - 34127 Trieste, Italy; 2 Laboratoire de Chimie Physique, CNRS, Université Paris-Sud, F-91405 Orsay, France; 3 Institut Charles Gerhardt, CNRS, Université de Montpellier, F-34095 Montpellier, France; 4 Department of Information Technology, University of Debrecen, H-4002 Debrecen, PO Box 400, Hungary; 5 Department of Theoretical Physics, University of Debrecen, H-4002 Debrecen, PO Box 400, Hungary; 6 ELI-ALPS, ELI-HU Non-Profit Ltd, Dugonics tér 13, H-6720 Szeged, Hungary

## Abstract

Recently we reported a series of numerical simulations proving that it is possible in principle to create an electronic wave packet and subsequent electronic motion in a neutral molecule photoexcited by a UV pump pulse within a few femtoseconds. We considered the ozone molecule: for this system the electronic wave packet leads to a dissociation process. In the present work, we investigate more specifically the time-resolved photoelectron angular distribution of the ozone molecule that provides a much more detailed description of the evolution of the electronic wave packet. We thus show that this experimental technique should be able to give access to observing in real time the creation of an electronic wave packet in a neutral molecule and its impact on a chemical process.

Since the advent of femtochemistry remarkable and decisive progress has been achieved on the experimental front and it is now possible to monitor electronic motion in the context of attophysics^1^,^2^,^3^,^4^. In other words, electronic wave packets can be created and observed in real time, which will improve our understanding of fundamental quantum concepts such as coherence and coherent light-matter interaction on the time scale of the electrons in a molecule.

Exciting molecules with attosecond XUV light pulses may populate several electronic states coherently, thus creating an electronic molecular wave packet. Its evolution will eventually trigger nuclear motion on a longer timescale via the effective potential created by the electrons and governing nuclear dynamics. In this context, a crucial challenge for attosecond sciences is to create specific electronic wave packets able to induce nuclear motion, e.g. a chemical process, selectively and efficiently. This should lead, on the long term, to what some already call attochemistry, where, at each step of a molecular process, the coupled motions of electrons and nuclei could be controlled on their natural time scales^5^. For example, if the attosecond pulse ionizes the molecule, the hole thus created will move, a process which is termed charge migration^5^. This may yield, in a second step, to selective bond dissociation^5^,^6^. Another possibility is to populate a limited number of electronic states in the neutral molecule by means of UV subfemtosecond pulses in order to trigger a selective chemical process. Experimentally, attosecond pulses are already available in the XUV spectral domain^7^ but few-cycle UV subfemtosecond pulses are expected to emerge in a near future.

A complete theoretical description of such processes is not a trivial task: it requires a quantum mechanical description of both the motion of the electrons and the nuclei in interaction with the external ultrafast field. In previous studies, we presented a full quantum mechanical simulation of the excitation of the ozone (neutral) molecule after excitation by a 3 fs UV pump pulse^8^,^9^,^10^,^11^. The central wavelength of the pulse at 260 nm was selected so as to create a coherent superposition of only two electronic states: the ground state, *X* (^1^*A*_1_), and the excited *B* (^1^*B*_2_) state^9^. The ozone molecule was chosen since, for obvious environmental reasons, its electronic excited states are well-known and understood^12^,^13^,^14^,^15^. In addition the *B* state is rather well isolated and, more importantly, the transition dipole between the *X* and *B* state is very large, leading to the so-called Hartley band in the UV domain that is responsible for the properties of the ozone layer. As a consequence, exciting the molecule to the *B* state does not require very high intensity (we used a value of 10^13^ W/cm^2^), and we can assume that only this state is populated by the laser pulse. However, it is worth noting that obtaining such intensities for very short UV pulses remains an experimental challenge at the moment.

In ref. 9, we investigated the creation of an electronic wave packet (see Fig. 6 in ref. 9) leading to an oscillation of the electronic charge density from one O-O bond to the other on the subfemtosecond time scale (with a period of 0.8 fs). This wave packet was thus an alternating superposition of two resonant forms that are precursors of the two dissociation channels O + O_2_ and O_2_ + O. Upon propagating nuclear wave packets with the Heidelberg Multi-Configuration Time-Dependent Hartree (MCTDH) package^16^,^17^,^18^,^19^,^20^,^21^, we showed that, at the end of the laser pulse, the molecule started to vibrate (see Fig. 4 in ref. 9). The quantum coherence between the two electronic states could thus be expected to be destroyed rapidly due to vibrations, even more so because of the dissociation outcome making this process irreversible. However, we observed a revival of coherence after the external field was off, with a time delay corresponding to a single vibrational period in the *B* state. This was attributed to a portion of the wave packet being trapped in the *B* state around a shallow potential energy well. Obviously, electronic coherence would have been preserved longer if the potential energy well of the *B* state had been deeper. In any case, this revival of quantum coherence is the signature that the coherent superposition of the two electronic states is not destroyed as soon as the nuclear motions starts. To conclude, we showed that it was possible to first create an electronic wave packet in the bound molecule, which would lead, in a second step, to the dissociation of the molecule and monitor the whole process with time-resolved spectroscopy. In principle, one could also expect to control this process upon manipulating the initial electronic wave packet via modulating the pump pulse.

From the experimental point of view, a wave packet cannot be observed as such, or at least not “directly” but rather from its consequences on the photodynamics of the system, via time-resolved observables obtained from pump-probe spectroscopy techniques. Attosecond XUV probe pulses can be used to ionize the molecule during the whole process with a time resolution compatible with the electronic motion^22^,^23^,^24^,^25^,^26^,^27^. The resulting time-resolved spectra from both electronic states, *X* and *B*, will provide precious information about the detailed dynamics of the system. Our probe pulse is centered around 95 eV. This high value generates electrons that are ejected with high velocities. A sudden approximation can thus be invoked to describe one-photon XUV ionization^28^. In addition, it is desirable that the ionization process is as instantaneous as possible so that it does not perturb the electronic motion induced by the pump pulse. In ref. 10, we calculated the relative ionization probabilities based on an approach exploiting Dyson orbitals (see ref. 10 for the calculation of these). Within the sudden approximation regime one can estimate relative cross sections as the square norms of the Dyson orbitals. Then, after convolution of the stick photoelectron spectra from *X* and *B*, we could calculate the time-resolved photoelectron spectrum (TRPES) as a function of time and photoelectron kinetic energy. This spectrum clearly exhibited depletion of *X* and production of *B*^11^.

Now, in order to analyze the wave packet created by the pump pulse in more detail, it is useful to consider a more accurate and complete description of the time-resolved photoelectron spectrum, including both realistic cross sections and angular distributions, and their photon energy dependence. For instance, molecular frame photoelectron angular distributions (MFPAD) give access to the shape of the electronic wave packet^24^. Even photoionization from molecules that are randomly distributed in terms of their orientation in space show important dependence on the angle between the polarization axis of the pump pulse and the direction of the ejected electron. The aim of the present work is precisely to provide such a time-resolved photoelectron angular distribution for the dissociation of ozone with the aforementioned pump pulse. This completes an ab-initio theoretical framework for the accurate description of pump-probe experiments in small molecules, represented here by O_3_, able to deal with electronic and nuclear motion on equal footing, describing the combined electron-nuclear wave packet.

The outline of the paper is as follows: in the next section we describe briefly the methods used for quantum chemistry calculations and quantum dynamics simulations. In the third section, the resulting photoelectron spectra are presented and discussed. Finally, conclusions provide an outlook for the future of molecular attophysics.

## Theoretical Background

A molecule such as ozone can be viewed as a collection of *N* nuclei and *n* electrons. Let 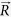 = 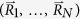 and 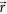 = 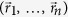 denote the position vectors of the nuclei and the electrons, respectively. Using a semi-classical approach with respect to the external electromagnetic field and the so-called dipole approximation, the non-relativistic Coulomb molecular Hamiltonian operator for the system interacting with a time-dependent external electric field, 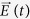, reads


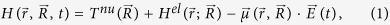


where 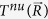 is the kinetic energy operator of the nuclei, 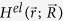 the electronic Hamiltonian operator (the sum of the latter two terms being the field-free molecular Hamiltonian), and 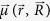 the electric dipole moment of the molecule.

The time-dependent Schrödinger equation reads


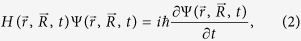


with 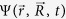 the wave packet of the molecule.

The adiabatic electronic basis functions, 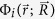, satisfy for each 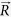



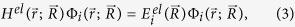


where 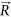 are to be viewed as parameters and 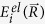 play the role of potential energy surfaces for the nuclei.

Here, we consider only a pair of adiabatic electronic states for ozone: *X*(^1^*A*_1_), the ground state, and *B*(^1^*B*_2_), the Hartley excited state. The total wave function of the molecule can be expanded as


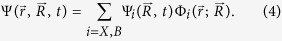


In the following, we assume the Born-Oppenheimer approximation to be valid and thus neglect the non-adiabatic couplings between the two electronic states stemming from the nuclear kinetic energy operator. The only coupling between *X* and *B* is induced by the external field through the term 
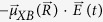
, where the transition dipole is defined as 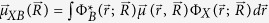. We also neglect the diagonal terms involving 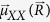 and 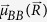 since 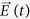 is an external field resonant between *X* and *B* with respect to the central wavelength of the spectrum of the pulse.

Thus, the evolution of 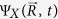 and 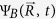 is governed by a set of two coupled equations involving only 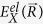, 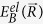, 
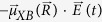
, and 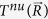. To solve this set of equations, i.e. to solve the Schrödinger equation for the nuclei, we use the MCTDH method^16^,^17^,^18^,^19^,^20^,^21^,^29^. The nuclear wave functions are expanded in a basis set of time–dependent functions, the so–called *single–particle functions* (SPFs),


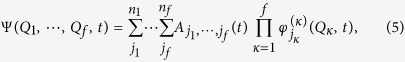


where *f* denotes the number of nuclear degrees of freedom (*Q*_*κ*_ are single coordinates or groups of coordinates involved in 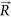). There are *n*_*κ*_ SPFs for the *κ*th nuclear degree of freedom. The equations of motion^16^,^17^,^18^,^19^,^20^,^21^ for the *A*-coefficients and the SPFs are derived from a variational principle that ensures optimal convergence.

In this work, *Q*_1_, ···, *Q*_3_ are (polyspherical) valence coordinates (*R*_1_ and *R*_2_, the two bond lengths, and *α*, the angle between the two bonds). The corresponding expression of the kinetic energy operator, *T *^*nu*^(*R*_1_, *R*_2_, *α*), with zero total angular momentum can be found in ref. 30. The potential energy surfaces, 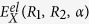 and 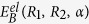, and the transition dipole surface, 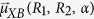, are those from Schinke and coworkers^13^,^14^,^15^. They are implemented in MCTDH and have already been tested on accurate applications in spectroscopy^31^,^32^,^33^,^34^.

**Figure 1:**
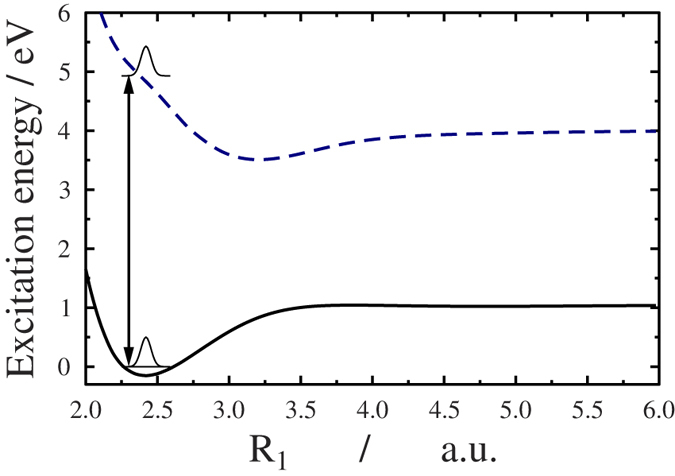
Potential energy cut of the ozone molecule as a function of the dissociation coordinate, *R*_1_: ground state (*X*, solid line) and Hartley state (*B*, dashed line), the arrow denotes the excitation of the *B* state. The other bond is fixed at *R*_2_ = 2.43 a.u. and the bond angle *α* = 117°.

The parameters defining 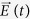, the laser pump pulse (see Fig. 1) are: central wavelength at 260 nm, intensity of 10^13^ W/cm^2^, Gaussian envelope with a full duration at half maximum (FDHM) equal to 3 fs. Note that, due to the *C*_2*v*_ symmetry of the ozone molecule at the Franck-Condon (FC) point (*R*_1_ = *R*_2_ = 1.275 Å; *α* = 116.9°), the *y*-component (*B*_2_) of the transition dipole between *X* and *B* is the only non-vanisihing one at the FC point and is thus primarily responsible for the light-induced electronic transitions. Consequently, the effective polarization axis of the electric field is *y*.

Further details regarding our calculations – the (time-independent) primitive basis sets, the parameters for the complex absorbing potentials, the refitting of the potential energy and transition dipole surfaces in a form adapted to MCTDH, and the number of SPFs – can be found in previous work, for instance in Sec. 3 of ref. 31.

Starting from the vibrational ground state in the electronic ground state *X*, MCTDH calculations will generate 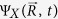 and 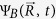 at any subsequent time. Assuming that only the *B* electronic state is populated by the laser pulse (see Fig. 1), the total molecular wave packet (see Eq. 4) can be constructed provided the corresponding adiabatic electronic wave functions are known.

Thus, with this approach, we can obtain in principle the full electronic and vibrational wave packet (note again that we only consider the case where the total angular momentum is equal to 0). However, this quantity cannot be observed directly in actual experiments and we need a time-resolved property that will characterize the time evolution of the system: the TRPES for instance, which can be measured and compared to calculations. The procedure that we used to compute this quantity is explained below.

As a first approximation, we can consider that the early stages of the process will be dominated by the behavior of the wave packet at the FC point, 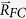. The corresponding renormalized density matrix of the molecule at the FC point (see Sec. II B of ref. 9 for further details) reads, for *i*, *i*′ = *X*, *B*,


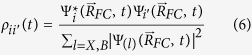


Note that such local populations of *X* and *B* are not classical quantities but extracted from the actual quantum wave packets.

Assuming a “stationary” picture, the approximate photoelectron spectra from either *X*^35^ or *B* at the FC point appear as stick spectra,


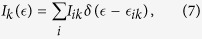


where 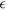 is the kinetic energy (KE) of the ejected electron, *i* = *X* or *B*, and *k* is used to label the various cation states. 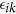 are the corresponding peaks appearing in the spectra. They satisfy


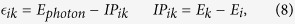


where *E*_*photon*_ denotes the energy of the probe photon, 95 eV here. *E*_*i*_ are the energies of the *X* and *B* states at the FC geometry, *E*_*k*_ the energies of the cation that can be populated by the photon at the same geometry, and *IP*_*ik*_ are the relative ionization potentials. Our calculations show that 19 cation states can be populated (up to about 20 eV above the *X* state)^11^. For the calculation of the peak intensities, *I*_*ik*_, we adopt an approach based on Dyson orbitals^10^. The latter are defined as


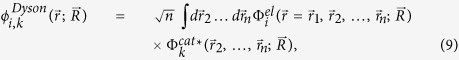


where 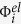 are the electronic functions of the neutral molecule as defined above and 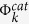 the electronic functions of the cation. We calculated Dyson norms at the FC point (see ref. 9) at the CASSCF(17,12)/aug-cc-pVQZ (no state average) level of theory for the cation wave functions and CASSCF(18, 12)/aug-cc-pVQZ (no state average) for the neutral wave functions with the MOLPRO quantum chemistry package^36^. The energies of the neutral and the cation were further refined with MRCI-SD(Q) calculations, including Davidson corrections, and based on the previous CASSCF references.

**Figure 2:**
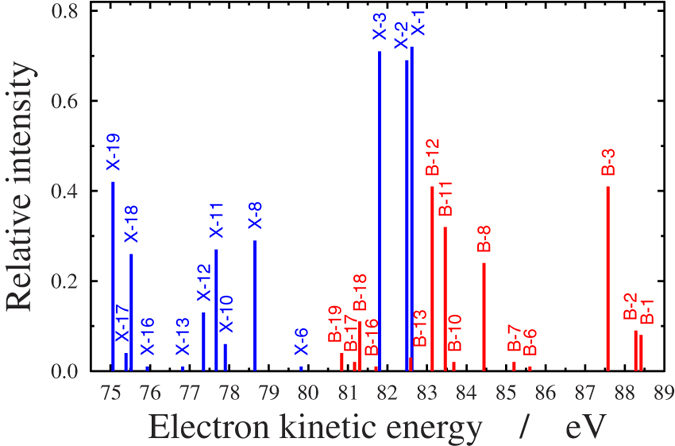
Stick photoelectron spectra from *X* (blue) or *B* (red) as functions of the energy of the ejected electron for a probe photon at 95 eV. Cation states (see Table 1) are labeled according to the order given in ref. 35; our calculations give *E*_15_ < *E*_14_ and *E*_18_ < *E*_17_, which is why *B* − 18 is before *B* − 17.

If a sudden approximation is assumed, the squares of the Dyson norms, 
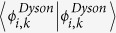
, are proportional to the relative ionization probabilities *I*_*ik*_. Ionization potentials and 
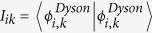
 are reported in 1. The corresponding stick spectrum is displayed in Fig. 2. To obtain the energy resolved spectra we convoluted the stick spectra with a Gaussian envelope function *G*(*ε*) to mimic the bandwidth of the XUV probe pulse,


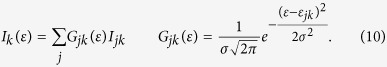


Here *σ* is the standard deviation of the intensity: *σ* = 1.5 eV for a probe pulse of FDHM equal to 500 as.

Let us now consider the full photoionization dynamics. Assuming a randomly oriented molecular sample, the differential cross section in the laboratory frame (LF) coordinate system is given by the following expression:


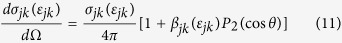


where 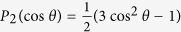 is the second order Legendre polynomials and *θ* is the angle between the direction of the electron momentum and the polarization of the electric field. Ω is the angle relative to electron emission momentum in the LF system and the two energy dependent parameters are *σ*_*jk*_ (partial cross section) and *β*_*jk*_ (asymmetry parameter). (The LF system defines the experiment i.e. the direction of the polarization and propagation of light as well as the direction of electron detection. The reference system is the molecular frame (MF) system in which the molecule is fixed and the electronic structure, transition dipole moment etc. calculations are performed.)

Calculation of *σ* and *β* parameters require an explicit description of the continuum wave function for the final state. Neglecting interchannel coupling effects, generally very small far from thresholds, a single channel approximation of the form


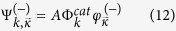


is generally quite accurate. Here 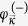 describes an electron with asymptotic momentum 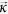 (and incoming wave boundary conditions, appropriate for photoionization), and *A* describes antisymmetrization and proper symmetry couplings. Actually it is computationally easier to work in an angular momentum basis, employing eigenstates


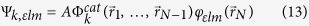


where the continuum wavefunctions *φ*_*εlm*_ are characterized by suitable asymptotic conditions, in our case K-matrix boundary conditions, defined as


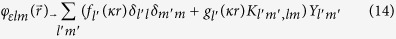


which has the advantage of working with real wave functions. Here *f*_*l*_ and *g*_*l*_ are regular and irregular coulomb functions. The *φ*_*εlm*_ so obtained can be transformed to incoming wave boundary conditions and then to linear asymptotic momentum by standard transformation^37^


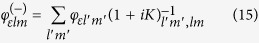



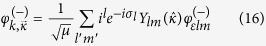


The same transformation can be directly applied to the transition dipole moments. The many-particle transition dipole moment


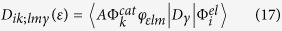


reduces to the single particle moment involving the Dyson orbital (9)


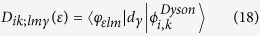


plus an additional term (conjugate term) which is generally small and is usually neglected^38^. Here *γ* is the Cartesian component of the dipole, *D* and *d* are the many-particle and the single particle dipole operators.

From dipole moments (and the *K*-matrix) *σ*_*jk*_(*ε*) and *β*_*jk*_(*ε*), as well as any angular distribution from oriented molecules, can be computed according to well known formulas^37^.

In our formulation, the continuum wave function (13) is computed as an eigenfunction of the Kohn-Sham Hamiltonian defined by the initial state electron density *ρ*


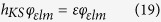



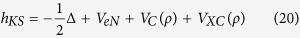


where *V*_*eN*_ is the nuclear attraction potential, *V*_*C*_ the coulomb potential and *V*_*XC*_ the exchange-correlation potential defined in terms of the ground state density *ρ*. The latter is obtained from a conventional LCAO SCF calculation, employing the ADF program with a DZP basis^39^,^40^. A special basis is employed for the continuum solutions of (19). Primitive basis functions are products of a *B*-spline radial function^41^,^42^ times a real spherical harmonic


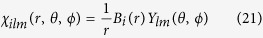


The full basis comprises a large one-center expansion on a common origin, with long range *R*_*max*0_, and large maximum angular momentum, *L*_*max*0_. This is supplemented by additional functions centered on the nuclei, of very short range, *R*_*maxp*_, and small angular momenta *L*_*maxp*_. A short range is necessary to avoid almost linear dependence of the basis, which spoils the numerical stability of the approach. Despite the very limited number of LCAO functions these choices ensure a very fast convergence of the calculated quantities. The basis is then fully symmetry adapted.

The calculation of continuum eigenvectors is performed at any selected electron kinetic energy by the Galerkin approach originally proposed in ref. 43 and the generalized to the multichannel case^44^,^45^. From the energy independent Hamiltonian *H* and overlap *S* matrices continuum vectors are obtained as eigenvectors of the energy dependent matrix *A*(*E*) = *H* − *ES* with eigenvalues very close to zero. These give the correct number of independent open channel solutions, and are efficiently obtained by block inverse iteration, since they are separated by large gaps from the rest of the spectrum. Actually the more stable form *A*^+^*A* is currently employed^46^. Final normalization to *K*-matrix boundary conditions is obtained by fitting the solutions to the analytical asymptotic form at the outer boundary *R*_*max*0_.

In the present calculation the LB94 *V*_*XC*_ potential^47^ was employed, due to the correct asymptotic behavior, important in photoionization. Parameters were *L*_*max*0_ = 12, *R*_*max*0_ = 25.0 a.u., with 135 B-splines of order 10, *L*_*maxp*_ = 2, *R*_*maxp*_ = 1.50 a.u. for the O atoms, for a total of 23013 basis functions.

Such an approach, called static-DFT proves in general remarkably accurate for the description of cross sections and angular distributions^41^,^48^,^49^. In conjunction with the Dyson orbital formulation it is able to describe ionization involving multiconfigurational initial and final cationic states^38^,^50^. We refer to previous work for details of the implementation^41^,^51^. *σ*_*jk*_ and *β*_*jk*_ are obtained on a dense electron KE *ε*_*jk*_ grid, so that the value at any KE dictated by the given photon energy can be accurately obtained by interpolation. With these the angularly resolved photoelectron intensity becomes:


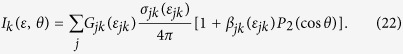


Applying the same convolution procedure as in Eq. 9 of ref. 11 we arrive to the appropriate formula of the angle resolved photoelectron spectrum:


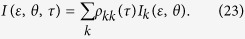


Here the *ρ*_*kk*_(*τ*) comes from eq. 6 and from now on the above expression (eq. 23) will serve as our working formula in the forthcoming part of the paper.

## Results and Discussion

**Figure 3:**
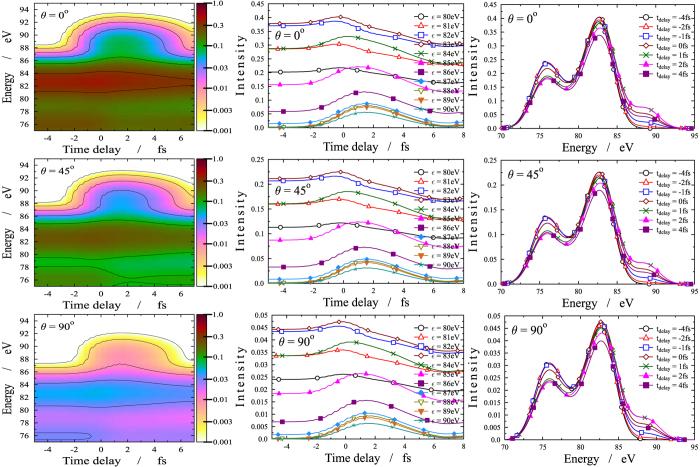
Angle resolved photoelectron spectrum (ARPES). First column: ARPES (logarithmic scale) as a function of the time delay (horizontal axis) and energy of the ejected electrons (vertical axis). The different panels correspond to different *θ* orientation angle (*θ* is the angle between the direction of the electron momentum and the polarization of the electric field). The intensity of the ejected electrons are coded by colors according to the scale on the right side. Second column: One dimensional cuts for the intensity of the ejected electrons via time delay with fixed *θ* and 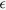. Third column: One dimensional cuts for the intensity of the ejected electrons via energy with fixed *θ* and *t*_*delay*_.

Figure 3 displays the intensity of the ejected electrons as a function of energy and time delay between the pump and probe pulses for three different fixed values of the orientation angle, *θ*. It can be seen that the ionization probability is larger for smaller angles. For *θ* > 45° it is drastically reduced. At early times, when *t*_*delay*_ < −2 fs, ionization can only take place from the ground state, *X*. Here, two clearly distinct high intensity bands are observed within the 75–78 eV and the 80–85 eV energy intervals. These are consistent with the large Dyson norms calculated between the *X* state of the neutral and some of the states of the cation (see Table 1). In particular, large Dyson norms are found between *X* and the 1st (0.72), 2nd (0.69), 3rd (0.71), 8th (0.29), 11th (0.27), 18th (0.26), and 19th (0.42) cationic states. The corresponding ionization potential values for these lie within (12.38–13.2) eV and (16.35–19.94) eV, thus resulting in two well separated energy regions, ~(80–85) eV and ~(75–78) eV. However, from *t*_*delay*_ = −2 fs on, the pattern becomes richer due to ionization appearing from *B* as well. The explicit consequence of this is a new band that appears around 88 eV in the *t*_*delay*_ = 0–4 fs time interval. This indicates that the *B* state starts to be populated, owing to the large value of the Dyson norm between *B* and the 3rd cationic state (0.41). In addition, significant ionization is achieved from *B* to the 8th (0.24), 11th (0.32), and 12th (0.41) cationic states, which corresponds to the energy band around (80–85) eV in the *t*_*delay*_ = 0–2 fs time interval. Simultaneously, for *t*_*delay*_ > 0 fs the *X* electronic state slowly depletes, thus providing fewer electrons ejected from the ground state, which results in smaller intensity values (see the color in the 75–78 eV energy region). The structure of the figures at larger angles (*θ* > 45°) are quite similar to the former ones, but the colors are much lighter due to lower intensities, reflecting that large orientation angles are much less likely to be involved efficiently in the ionization.

**Figure 4:**
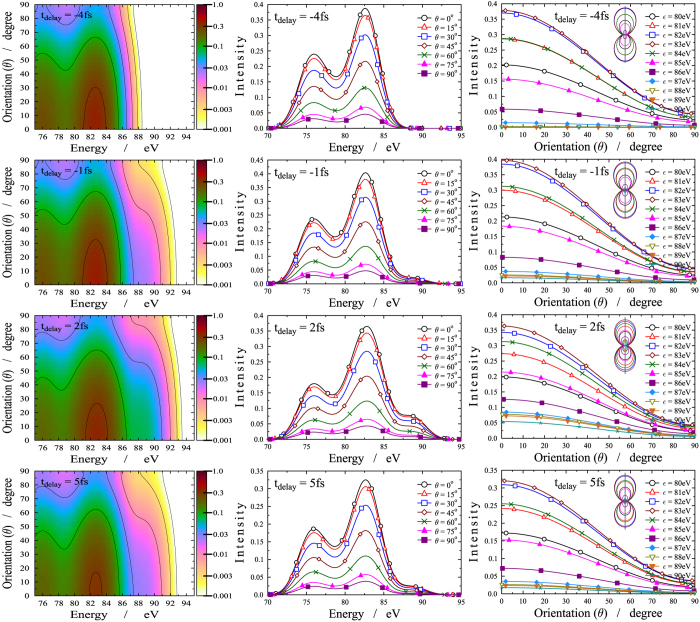
Angle resolved photoelectron spectrum (ARPES). First column: ARPES (logarithmic scale) as a function of the energy of the ejected electrons (horizontal axis) and orientation angle *θ* (*θ* is the angle between the direction of the electron momentum and the polarization of the electric field) (vertical axis). The different panels correspond to different time delays between the pump and probe pulses. The intensity of the ejected electrons are coded by colors according to the scale on the right side. Second column: One dimensional cuts for the intensity of the ejected electrons via energy with fixed *t*_*delay*_ and *θ*. Third column: One dimensional cuts for the intensity of the ejected electrons via electron emission orientation with fixed *t*_*delay*_ and 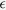.

**Figure 5:**
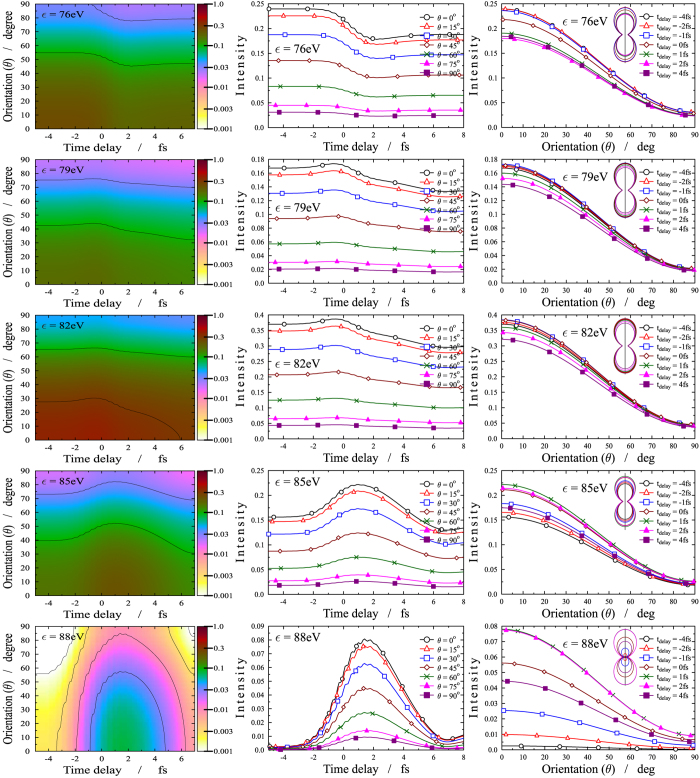
Angle resolved photoelectron spectrum (ARPES). First column: ARPES (logarithmic scale) as a function of the time delay *t*_*delay*_ (horizontal axis) and orientation angle *θ* (*θ* is the angle between the direction of the electron momentum and the polarization of the electric field) (vertical axis). The different panels correspond to different energies of the ejected electrons. The intensity of the ejected electrons are coded by colors according to the scale on the right side. Second column: One dimensional cuts for the intensity of the ejected electrons via time delay with fixed 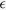 and *θ*. Third column: One dimensional cuts for the intensity of the ejected electrons via electron emission orientation with fixed *E* and *t*_*delay*_.

The above findings are confirmed on Figs 4 and 5, where the same results are presented differently. On Fig. 4, the electron emission orientation is given against the energy of the ejected electrons at several consecutive times. We observe that, up to *t*_*delay*_ = −1 fs, only two energy regions, (75–78) eV and (81–84) eV, exhibit significant intensity. They correspond to ionization taking place from *X* only. Ionization occurring from *B*, once *t*_*delay*_ > −2 fs, is characterized by the third band that appears around 88 eV and disappears slowly beyond *t*_*delay*_ > 4 fs. Within the *t*_*delay*_ = 1–2 fs time interval, the strengthening of the middle band reflects the combined impact of ionization occurring from both states together. Again, one clearly sees that, as a general trend, the intensity decreases monotonically as the angle between the ejected electrons and the direction of the polarization increases.

In Fig. 5, the electron emission orientation is plotted as a function of the time delay for several fixed electron energy values. Again, one observes large intensities in the (75–77) eV energy region and *t*_*delay*_ < 0 fs time interval for small orientation angles. The latter corresponds to the lack of population of the *B* state resulting in ionization taking place only from the *X* state. For *t*_*delay*_ > 0 fs, the decrease of the intensity indicates depletion of the *X* state. For 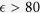 eV, a joint effect of ionizations from *X* and *B* is observed, more substantially from *X*. Again, the shape and the structure of the band for 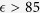 eV and *t*_*delay*_ = (−2)–6 fs is typical of ionization occurring from *B*.

From Fig. 5 it also appears that the angular distribution is strongly peaked along the probe field polarization, which is consistent with a high *β* value, close to two, for all ionizations. This is not surprising because of the high photon energy of the probe, 95 eV, which implies high kinetic energy of the outer valence ionized electrons, typically characterized by high *β* values, similar for all ionizations.

Finally the oscillatory patterns appearing in Figs 3 and 5 are clear fingerprints of the time dependence of the external electric field. Specifically, the pump pulse is a few-cycle pulse of width 3 fs and period 0.87 fs, centered around 260 nm (4.8 eV) in the deep UV (UV-C) domain and therefore its oscillation is faster than the nuclear motion.

In summary, the most representative signal is perhaps the upper-right panel in Fig. 3 (intensity against electron kinetic energy at different time delays for *θ* = 0°). It is clear that the largest temporal change in the spectrum is associated with the highest kinetic energies, from 86 to 89 eV, which are exclusively emitted from the *B* state, where the intensity increases significantly just after the pump pulse. Correspondingly, the decrease of the intensity after the pump is most evident in the low kinetic energy region, from 75 to 78 eV, due to the depleting of the *X* state, which is the dominant contribution in this energy window.

## Conclusions

A numerical simulation protocol has been developed for describing the electron dynamics of the ozone molecule in the Franck-Condon region involving only the ground (*X*) and Hartley (*B*) electronic states in the dynamics. Assuming isotropic initial distribution for the molecular ensemble, angle resolved photoelectron spectra have been calculated for various time delays between the pump that creates the wave packet (coherent superposition of *X* and *B*) and the probe that ionizes from either *X* or *B*. This physical quantity can be measured in actual experiments and compared to our calculations.

The present results are very encouraging and call for further improvements concerning the accuracy of the dynamics simulations. Therefore, our future aim is to perform more realistic simulations upon going beyond the presently assumed limiting hypotheses: isotropic initial distribution and populations extracted at the FC geometry only. This will be manifested by two significant changes in the numerical protocol: *i)* after the pump pulse is off alignment of the molecular ensemble will be assumed; *ii)* instead of performing calculations at a single FC geometry, several other nuclear geometries will be involved in the FC region where the nuclear density has significant value too.

We stress again that given the dipole matrix elements and K-matrix, all photoionization observables can be computed, like photoionization from fixed-in-space molecules (MFPADS) or partially oriented molecules, as well as suitable averages over final detector energy and angle resolution^49^, to accurately describe any specific experimental setup. Actually the 95 eV pulse employed in the present study was suggested by an experimental colleague. With hindsight angular distribution from unoriented molecules turn out not to be very informative, given the *β* values close to 2 for all final states at this relatively large photon energy. Working at lower energies would produce larger anisotropies. Moreover working with oriented molecules, which is a goal actively pursued in such studies, would further much enhance anisotropies, different for each initial and final state.

The present numerical simulations clearly indicate that angle and time resolved photoelectron spectra can be used in molecular attophysics to characterize the creation of an electronic wave packet in a neutral molecule on the subfemtosecond time scale. We expect our computational study to be followed by experiments showing similar results.

As the number of experimental choices is quite large, we found it important to set up a fully ab-initio general formulation that will accommodate any specific experimental setup. We look forward to upcoming experiments to validate the theoretical framework provided here.

## Additional Information

**How to cite this article**: Decleva, P. *et al*. Attosecond electronic and nuclear quantum photodynamics of ozone monitored with time and angle resolved photoelectron spectra. *Sci. Rep*. **6**, 36613; doi: 10.1038/srep36613 (2016).

**Publisher’s note:** Springer Nature remains neutral with regard to jurisdictional claims in published maps and institutional affiliations.

## Figures and Tables

**Table 1:** *Ab initio* ionization potentials (MRCI-SD(Q) level of theory) and *I*_*ik*_, the squares of the Dyson norms (CASSCF/aug-cc-pVQZ level of theory) with respect to either *X* or *B* at the FC point.

	Cation states (*j*)	*Ej* − *EX*/eV	*Iik*(*X*)	*Ej* − *EB*/eV	*Iik*(*B*)
1	(1^2^*A*_1_)	12.38	0.72	6.59	0.08
2	(1^2^*B*_2_)	12.51	0.69	6.72	0.09
3	(1^2^*A*_2_)	13.20	0.71	7.42	0.41
4	(1^2^*B*_1_)	14.14	0.00	8.36	0.00
5	(2^2^*A*_2_)	14.45	0.00	8.66	0.00
6	(2^2^*B*_2_)	15.18	0.01	9.40	0.01
7	(2^2^*A*_1_)	15.58	0.00	9.80	0.02
8	(2^2^*B*_1_)	16.35	0.29	10.56	0.24
9	(3^2^*A*_2_)	16.50	0.00	10.72	0.00
10	(3^2^*B*_1_)	17.10	0.06	11.32	0.02
11	(3^2^*A*_1_)	17.33	0.27	11.54	0.32
12	(3^2^*B*_2_)	17.65	0.13	11.87	0.41
13	(4^2^*B*_2_)	18.18	0.01	12.41	0.03
14	(4^2^*A*_2_)	18.64	0.00	12.85	0.00
15	(4^2^*B*_1_)	18.61	0.00	12.83	0.00
16	(4^2^*A*_1_)	19.07	0.01	13.29	0.01
17	(5^2^*B*_2_)	19.61	0.04	13.83	0.02
18	(5^2^*A*_1_)	19.48	0.26	13.70	0.11
19	(6^2^*B*_2_)	19.94	0.42	14.16	0.04

The energy difference between the *X* and *B* states is 5.78 eV. (Experimental ionization potentials and further theoretical values can be found for comparison in refs 35, 39 and 40).
